# The Value of High‐Frequency Ultrasound and Color Doppler Flow Imaging in Assessing the Efficacy of Moderate‐to‐Severe Acne Vulgaris: A Prospective Single‐Arm Study

**DOI:** 10.1111/jocd.70793

**Published:** 2026-03-31

**Authors:** Litao Zheng, Xiaogang Fu, Xiaoying Qiu, Song Gao, Yaqun Jiang, Xue Wang, Kashif Ali, Fangru Chen

**Affiliations:** ^1^ Center of Burns and Plastic Surgery and Dermatology The 924th Hospital of Joint Logistics Support Force of the Chinese People's Liberation Army (PLA) Guilin China; ^2^ Department of Dermatology The First Affiliated Hospital of Guilin Medical University Guilin China; ^3^ Department of Dermatology The First People's Hospital of Yulin Yulin China; ^4^ Department of Ultrasound The First Affiliated Hospital of Guilin Medical University Guilin China; ^5^ School of International Education Guilin Medical University Guilin China

**Keywords:** acne vulgaris, color Doppler flow imaging, high‐frequency ultrasound

## Abstract

**Objective:**

This study aimed to investigate the value of non‐invasive skin detection techniques for the therapeutic assessment of moderate‐to‐severe acne vulgaris.

**Methods:**

In this study, we collected patients with moderate‐to‐severe acne vulgaris who attended the Department of Dermatology at the First Affiliated Hospital of Guilin Medical University between October 2021 and December 2022, and analyzed their high‐frequency ultrasound (HFUS) and color Doppler flow imaging (CDFI) characteristics at the Weekend 0 (T0), Weekend 4 (T1), and Weekend 12 (T2) of their treatment.

**Results:**

A total of 30 patients with moderate‐to‐severe acne vulgaris [mean age 21.1 ± 3.4 years; 14 males (46.7%), 16 females (53.3%)] were enrolled. HFUS assessment revealed improvements (*p* < 0.05) in multiple parameters following treatment. Prior to treatment, there was perfect agreement (*κ* = 1.0) between the Pillsbury clinical grading and the SSSA ultrasound grading in all 30 patients (100%). Among the 30 patients who completed the 12‐week follow‐up, post‐treatment ultrasound grading was higher than the clinical Pillsbury grading in 17 cases (56.7%). Agreement between the two grading methods post‐treatment was poor (*κ* = 0.3, *p* = 0.645, 95% CI: −0.17, 0.77), though statistical significance was limited by sample size.

**Conclusion:**

HFUS effectively visualizes deep acne lesions (nodules/cysts). Although ultrasound grading demonstrates slower severity reduction than clinical assessment, it provides more objective severity evaluation. CDFI enhances hemodynamic analysis through real‐time flow visualization. Combined HFUS/CDFI enables enhanced severity and prognostic assessment in moderate‐to‐severe acne vulgaris.

## 1 Introduction

Acne vulgaris is a chronic inflammatory disease of the pilosebaceous unit, ranks as the world's eighth most prevalent disease with a global cross‐age prevalence of 9.38% [1]. The clinical manifestations of acne vulgaris are pleomorphic lesions including comedones, inflammatory papules, pustules, nodules, cysts, and scarring, with inflammation being central to its pathogenesis and progression [2]. Its pathogenesis is currently thought to be related to the relative elevation of androgens, increased sebum production, perifollicular hyperkeratosis, and colonization by *Propionibacterium acnes* ( *P. acnes* ). In moderate‐to‐severe cases, inadequate or delayed treatment frequently leads to scarring and post‐inflammatory hyperpigmentation. These sequelae significantly impair quality of life and are associated with elevated risks of anxiety, depression, and suicidal ideation [3, 4, 5]. Consequently, precise severity assessment is essential for personalized therapeutic strategies. While the International Modified Classification (Pillsbury Classification) remains widely employed [6], its dependence on subjective clinical evaluation limits accurate assessment of microscopic and deep structural lesions, failing to meet contemporary clinical demands. An objective, reproducible assessment methodology is therefore urgently needed.

HFUS typically defined as ultrasound utilizing probe frequencies ≥ 15 MHz, enables detailed visualization of skin architecture, including depth, thickness, and local perfusion [7]. As a rapid, safe, and non‐invasive imaging modality, HFUS provides objective assessments crucial for the diagnosis, monitoring, and management of dermatological conditions. Beyond accurately measuring epidermal and dermal thickness, HFUS excels in delineating the dimensions and depth of deeper cutaneous lesions [8]. Specifically, in acne vulgaris, it facilitates the evaluation of deep inflammatory components such as nodules/cysts and hyperechoic within cysts, etc., thereby offering valuable insights into disease severity and prognosis. Complementary to HFUS, CDFI integrates the Doppler effect with conventional B‐mode ultrasound to visualize blood flow direction and velocity in real‐time, depicting hemodynamic information as color‐coded signals [9]. This capability allows for the direct visualization and assessment of blood flow alterations within inflammatory foci, serving as an indicator of inflammatory activity. In summary, this study aimed to investigate the clinical features, influential factors, and pre‐ and post‐treatment characteristics of HFUS and CDFI in patients with moderate‐to‐severe acne vulgaris, with the goal of providing a reliable auxiliary tool for clinical efficacy assessment.

## 2 Methods

### 2.1 Study Population

The study enrolled patients with moderate‐to‐severe acne vulgaris attending the outpatient dermatology clinic at the First Affiliated Hospital of Guilin Medical University between October 2021 and December 2022.

The inclusion criteria: (1) Meeting the diagnostic criteria for acne vulgaris [10]; (2) Clinical grading using the International Modified Grading System (Pillsbury Grading System) to assess the severity of acne vulgaris; HFUS grading was based on the Sonographic Scoring System for Acne (SSSA) method [11]; (3) Provision of written informed consent; (4) Adequate literacy to independently complete study questionnaires (or with minimal assistance) and able to complete the follow‐up observation as required (5) Age between 13 and 40 years (inclusive).

Exclusion Criteria: (1) Presence of concomitant facial dermatoses; (2) Drug‐ or chemical‐induced acne vulgaris; (3) Significant psychiatric disorders, pregnancy, or lactation; (4) Inability to cooperate with questionnaire completion or non‐invasive testing procedures.

### 2.2 Study Design

This prospective, single‐center study collected clinical data including age at onset, gender, body mass index (BMI), ethnicity, occupation, education level, predominant lesion type, disease duration, season of onset, and skin type. All patients received a standardized regimen of either oral isotretinoin, doxycycline, or clarithromycin, combined with topical clindamycin phosphate gel. Based on individual economic circumstances, patients could additionally opt for one of two adjunctive physiotherapy modalities: the Brilliance 360 photonic system (640 nm pulsed light) or photodynamic therapy. Patients were followed up at the 4th and 12th weeks of treatment. Treatment regimens remained unchanged throughout the study period.

### 2.3 Skin Assessment

To compare clinical efficacy before and during treatment, all target skin lesions were assessed using standardized clinical evaluation combined with HFUS and CDFI. Assessments were performed at three predefined time points: baseline (T0, prior to treatment initiation), week 4 (T1), and week 12 (T2) of the treatment period. In our study, both HFUS and clinical assessments were independently performed by different specialists (ultrasonologists and dermatologists), with both parties blinded to each other's results.

#### 2.3.1 Digital Photo Capture

Standardized digital photography of skin lesions was performed at all assessment time points (T0, T1, T2) by the same board‐certified dermatologist using an identical digital single‐lens reflex (DSLR) camera (Canon 750D, Japan). To ensure consistency and comparability across serial images, photographs were captured under rigorously controlled conditions, maintaining identical camera angles, standardized ambient lighting, and a stable environmental temperature for each patient.

#### 2.3.2 HFUS, CDFI Image Acquisition and Analysis

HFUS imaging was conducted on patients with moderate‐to‐severe acne vulgaris by a certified sonographer using a Hitachi Aloka ARIETTA 70 color Doppler ultrasound system equipped with a variable frequency linear array transducer (5–18 MHz). Adhering to a standardized protocol, patients were positioned supine or laterally to fully expose the target facial regions (forehead, cheeks, chin, nose). Following application of an adequate amount of medical ultrasound coupling gel, the transducer frequency was set to 18 MHz and positioned perpendicular to the skin surface. Minimal transducer pressure was applied to avoid tissue deformation, confirmed by the absence of visible skin indentation. Lesions within each predefined facial area were systematically identified. For each area, the single most severely affected lesion, as determined by the interpreting ultrasound physician, was selected for detailed analysis: (1) Region Identification: The entire face was systematically scanned using HFUS. The region exhibiting the highest density of acne lesions and the most extensive inflammatory involvement (e.g., cheek, forehead, nose) was objectively identified. (2) Lesion selection: Lesion selection within this region followed a hierarchical protocol: the lesion with the largest transverse diameter was selected first. For lesions of comparable diameter, the one with the greater depth was prioritized. Furthermore, when these morphological parameters were similar, a Color Doppler flow signal grade ≥ II (i.e., exhibiting 3–4 punctate signals or one major vessel) served as the tie‐breaking criterion to ensure the inclusion of lesions with higher inflammatory activity. At each time point (T0, T1, T2), the following parameters were assessed in this target lesion: dermal thickness; presence and characteristics of perifollicular echogenicity changes; size (maximum diameter) and depth of the largest pseudocyst; presence of significant epidermal elevation; evidence of lesion fusion (coalescence); presence of intracystic hyperechoic foci (suggestive of calcification); and signs of subcutaneous fat layer involvement (e.g., disruption of the dermal‐subcutaneous junction or hypoechoic changes within the fat). All selections were performed by one of three sonographers, each with over 5 years of experience in dermatologic ultrasound. Crucially, every selection was independently verified by a second, equally qualified sonographer. The entire process was documented in the ultrasound reporting system to ensure traceability and minimize subjective bias.

Subsequently, CDFI was performed to assess peri‐lesional vascularity according to the Adler grading system [12], as follows: Grade 0 (avascular): no detectable flow; Grade I (minimal): 1–2 punctate signals; Grade II (moderate): 3–4 punctate signals or one major vessel; Grade III (marked): ≥ 5 punctate signals or multiple vessels. Standard Doppler settings included a pulse repetition frequency of 800 Hz and optimized gain to suppress noise. All acquired HFUS and CDFI images, corresponding measurement values, and qualitative assessments were securely stored digitally. Data were systematically recorded, linked to the specific patient, facial area, target lesion, and time point to ensure accurate longitudinal tracking of identical regions.

### 2.4 Statistical Analyses

Statistical analyses were performed with SPSS 28.0 software. Continuous variables are presented as mean ± standard deviation (SD) for normally distributed data, and as median (P25, P75) for non‐normally distributed data. Categorical data are presented as frequency (%) and compared between groups using the Chi‐square (*χ* ^2^) test. Non‐parametric rank‐sum tests were used for ordinal dependent variables. Ordinal variables were analyzed using nonparametric tests. Overall differences in Adler blood flow grading at weeks 0, 4, and 12 among the 30 patients were assessed using the Friedman M test. When the overall test was significant, pairwise comparisons were performed using the Wilcoxon signed‐rank test with Bonferroni correction applied (adjusted *α* = 0.017). Acne severity was evaluated using the Pillsbury grading system and the SSSA grading system. Agreement between the two methods was assessed using Cohen's kappa (*κ*) coefficient with 95% confidence intervals (CIs). Agreement strength was defined as high (*κ* ≥ 0.75), moderate (0.40 ≤ *κ* < 0.75), or poor (*κ* < 0.40). The statistical significance of *κ* was determined using a z‐test (null hypothesis: *κ* = 0). All tests were two‐tailed, and *p* < 0.05 was considered statistically significant, except for pairwise comparisons (*p* < 0.017).

## 3 Results

### 3.1 Clinical Characterization of Patients With Moderate‐to‐Severe Acne Vulgaris

A total of 30 patients with moderate‐to‐severe acne vulgaris were enrolled in this study. The mean age was 21.1 ± 3.4 years. There were 14 male patients (46.7%) and 16 female patients (53.3%), yielding a male‐to‐female ratio of 1:1.14. The mean body mass index (BMI) was (19.6 ± 2.3) kg/m^2^, of which 17 patients (56.7%) had a BMI of 18.5–24.9 kg/m^2^. Disease activity showed no seasonal variation. Oily skin type was predominant, observed in 76.7% of patients (Tables S1 and S2).

### 3.2 HFUS Characterization of Patients With Moderate‐to‐Severe Acne Vulgaris Before and After 4 and 12 Weeks of Treatment

There were statistically significant differences in terms of the largest nodule/cyst length diameter, the largest nodule/cyst width diameter, its depth from the body surface, its hyperechoic length diameter, dermal thickness at the lesion site, normal dermal thickness, and epidermal elevation at the three time points of T0, T1, and T2 (*p* < 0.001). The differences in skin fusion, strong echo in cyst, and fat layer involvement were statistically significant (*p* < 0.05) (Table 1).

**TABLE 1:** HFUS characterization of patients with moderate‐to‐severe acne vulgaris before and after 4 and 12 weeks of treatment.

	Duration of treatment			*Z/χ* ^2^	*p*
	T0 (mm, number, %)	T1 (mm, number, %)	T2 (mm, number, %)		
Largest nodule/cyst length diameter	7.00 (5.38, 10.60)	5.85 (4.08, 9.45)	3.30 (2.60, 5.45)	38.235^a^	< 0.001
Largest nodule/cyst width diameter	2.75 (2.38, 3.63)	2.65 (1.88, 2.83)	1.70 (1.25, 2.03)	32.814^a^	< 0.001
Distance of the base of the largest cyst from the body surface	3.20 (2.48, 4.30)	2.60 (2.08, 3.00)	1.90 (1.68, 2.48)	41.542^a^	< 0.001
largest cyst hyperechoic length diameter	1.35 (0.98, 1.63)	0.90 (0.60, 1.53)	0.80 (0.58, 1.15)	30.336^a^	< 0.001
Dermal thickness at the largest cyst	3.30 (2.28, 4.35)	2.60 (2.18, 2.85)	1.80 (1.40, 2.23)	28.632^a^	< 0.001
Normal dermal thickness	1.70 (1.40, 2.05)	1.45 (1.28, 1.60)	1.25 (1.10, 1.40)	26.895^a^	< 0.001
Epidermal eminence					
Yes	28 (93.3)	19 (63.3)	10 (33.3)	22.995^b^	< 0.001
No	2 (6.7)	11 (36.7)	20 (66.7)		
Skin lesion fusion					
Yes	19 (63.3)	16 (53.3)	9 (30.0)	6.596^b^	0.010
No	11 (36.7)	14 (46.7)	21 (70.0)		
Hyperechoic in cysts					
Yes	24 (80.0)	20 (66.7)	12 (40.0)	10.097^b^	0.001
No	6 (20.0)	10 (33.3)	18 (60.0)		
Fat layer involvement					
Yes	24 (80.0)	23 (76.7)	14 (46.7)	7.547^b^	0.006
No	6 (20.0)	7 (23.3)	16 (53.3)		

*Note:* T0, T1, T2: *M* (*P*25, *P*75).

^a^ Friedman *M*‐test.

^b^ Linear trend chi‐square test.

### 3.3 Analysis of the Number of Manual and HFUS Nodules/Cysts in Moderate‐to‐Severe Acne Vulgaris After 4 and 12 Weeks of Treatment

Manual and HFUS assessments of nodule and cyst counts in patients with moderate‐to‐severe acne vulgaris demonstrated statistically significant differences at all three time points (T0, T1, T2) (*p* < 0.001, Table 2).

**TABLE 2:** Differences between manual and HFUS for nodule/cyst counts before and after 4 and 12 weeks of treatment for moderate‐to‐severe acne vulgaris.

Detection methods	T0	T1	T2	*Z*	*p*
Manual counting	10 (6, 20)	7.5 (4, 14.25)	3 (2, 7.25)	−7.315	< 0.001
HFUS	20 (15.75, 40)	20 (13, 31.25)	12 (7.75, 20)		

*Note:* T0, T1, T2: *M* (*P*25, *P*75).

### 3.4 Analysis of Adler Grading of Blood Flow in Moderate to Severe Acne Before and After Treatment at 4 and 12 Weeks

Friedman's test revealed significant differences in Adler grading of blood flow among the 30 patients at weeks 0, 4, and 12 (*M* = 36.587, df = 2, *p* < 0.001, Table 3), with a gradual decreasing trend over time. Pairwise comparisons using the Wilcoxon signed‐rank test (Bonferroni correction, *α* = 0.017) showed significant differences between week 0 and week 12, and between week 4 and week 12 (both *p* < 0.001); no significant difference was found between week 0 and week 4 (*p* = 0.024 > 0.017). Based on the above, the blood flow characteristics of CDFI can reflect the inflammatory state of acne lesions, providing an objective basis for clinical evaluation.

**TABLE 3:** Comparison of moderate to severe acne before and after treatment (at 4 and 12 weeks) with Adler grading of blood flow.

	T0	T1	T2	*M*	*p*
Adler grade 0	1	1	4	36.587	< 0.001
Adler grade 1	3	8	15		
Adler grade 2	3	3	6		
Adler grade 3	23	18	5		

### 3.5 Consistency Analysis of Clinical and Ultrasound Assessments Before and After 12 Weeks of Treatment

Baseline clinical staging showed perfect agreement with SSSA ultrasound staging in all 30 patients (*κ* = 1.00, *p* < 0.001, 95% CI: 1.00, 1.00), indicating excellent pre‐treatment concordance (Table 4).

**TABLE 4:** Compliance rates with SSSA/Pillsbury grading before treatment for moderate to severe acne vulgaris.

SSSA/Pillsbury grading	Pillsbury mild	Pillsbury moderate	Pillsbury severe	Total (%)
SSSA grade I	0	0	0	0 (0)
SSSA grade II	0	3	0	3 (20.0)
SSSA grade III	0	0	27	27 (90.0)
Total (%)	0 (0)	3 (10.0)	27 (90.0)	30 (100)

**FIGURE 1:**
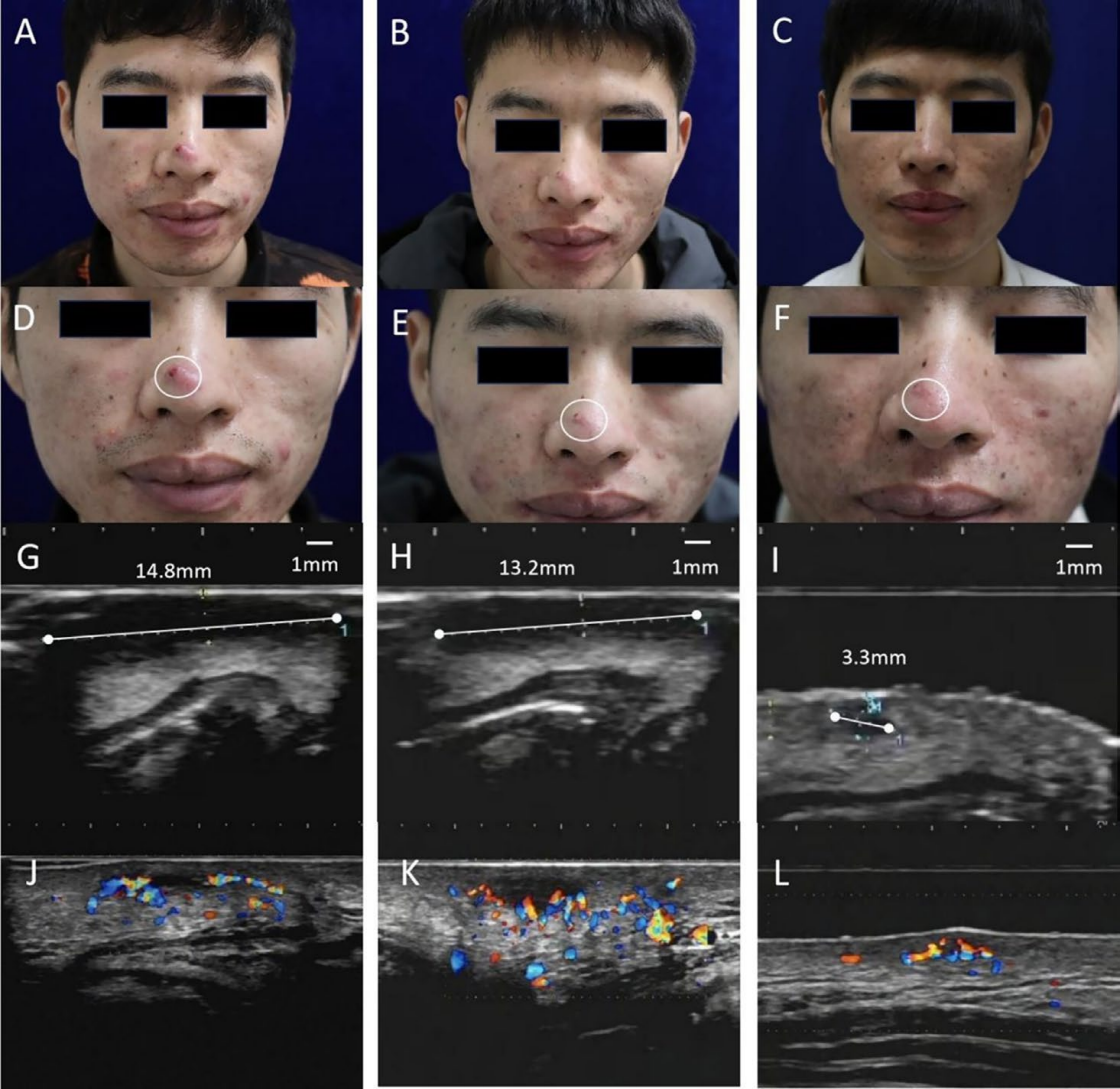
Clinical, ultrasonographic, and Doppler findings in a 20‐year‐old male with moderate‐to‐severe acne vulgaris. Clinical progression: (A) Baseline: Facial presentation showing inflammatory papules, pustules, cysts, and scarring. (B) Week 4: Reduced papule and pustule count with persistent cysts. (C) Week 12: Significant clinical improvement; minimal residual erythematous papules and scarring. Severest lesion evolution & Pillsbury grading: (D) Baseline: Large cyst (white circle); Pillsbury grade: Severe. (E) Week 4: Most severe lesion: Inflammatory papule (white circle); Pillsbury grade: Moderate. (F) Week 12: Most severe lesion: Scar (white circle); Pillsbury grade: Mild. HFUS (18 MHz) findings: (G) Baseline: Dermal‐subcutaneous pseudocyst (14.8 × [width] mm; > 5 mm depth), extending deep to adipose layer with indistinct borders and posterior acoustic enhancement (SSSA Grade III). (H) Week 4: Persistent pseudocyst (13.2 × [width] mm; SSSA Grade III). (I) Week 12: Significant resolution; residual focal dermal hypoechoic focus (3.3 × [width] mm; SSSA Grade II). CDFI: (J) Baseline: Marked intralesional vascularity. (K) Week 4: Persistent abundant vascularity. (L) Week 12: Minimal residual vascular flow.

**FIGURE 2:**
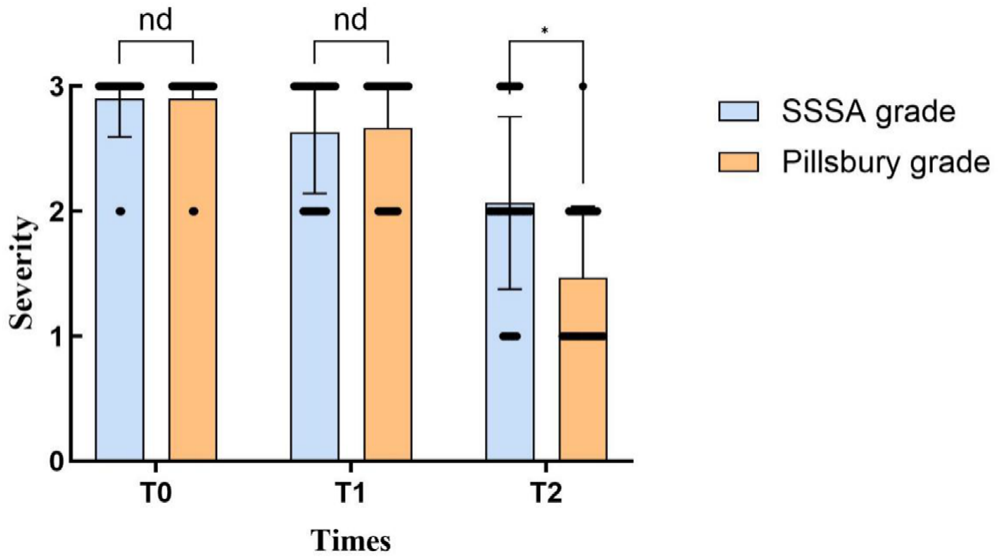
Comparison of SSSA and Pillsbury classification, before and after treatment. With the *x*‐axis representing the duration of treatment and the y‐axis representing the severity (1: Mild; 2: Moderate; 3: Severe).

Following 12 weeks of treatment, HFUS classified patients as SSSA‐I (*n* = 5, 16.7%), SSSA‐II (*n* = 17, 56.7%), and SSSA‐III (*n* = 8, 26.7%). In contrast, clinical Pillsbury grading assigned mild (*n* = 16, 53.3%), moderate (*n* = 12, 40.0%), and severe (*n* = 2, 6.7%) classifications. Concordance between methods was observed in 11 patients (36.7%). Discordant results occurred in 19 patients (63.3%), with ultrasonographic grading exceeding clinical grading in 17 cases (56.7% of cohort, Figure 1). Overall agreement between post‐treatment Pillsbury and SSSA grading was poor (*κ* = 0.30, *p* = 0.645, 95% CI: −0.17, 0.77) and statistically non‐significant (Figure 2). This indicates diminishing concordance with treatment duration, with ultrasonography consistently detecting greater disease severity than clinical assessment in most patients (Table 5).

**TABLE 5:** Conformity of SSSA/Pillsbury grading after 12 weeks of treatment for moderate‐to‐severe acne vulgaris.

SSSA/Pillsbury grading	Pillsbury mild	Pillsbury moderate	Pillsbury severe	Total (%)
SSSA grade I	4	1	0	5 (16.7)
SSSA grade II	10	6	1	17 (56.7)
SSSA grade III	2	5	1	8 (26.7)
Total (%)	16 (53.3)	12 (40.0)	2 (6.7)	30 (100)

## 4 Discussion

Acne vulgaris is a chronic inflammatory disease with peak incidence between the ages of 15 and 20 [13]. Treatment of moderate‐to‐severe disease, characterized by cysts, nodules, and inflammation, is often prolonged. Untreated, it carries significant risks of scarring and psychological sequelae, including diminished self‐esteem, depression, and anxiety [14]. Premature treatment discontinuation in some patients readily leads to recurrence, potentially due to incomplete clearance of deep‐seated cysts and nodules. Repeated and extended treatment courses impose substantial burdens on patients. Consequently, accurate severity assessment and timely therapeutic adjustments during management are imperative.

Currently, clinical assessment of moderate‐to‐severe acne vulgaris primarily relies on visual observation and palpation. This subjective approach can result in premature treatment cessation before deep‐seated lesions are fully resolved, leading to recurrence and contributes to inter‐observer variability, hindering consistent treatment outcomes.

This study compared agreement between manual clinical (Pillsbury) and HFUS (SSSA) assessments pre‐ and post‐treatment. Pre‐treatment agreement was perfect in all 30 cases (*κ* = 1). At 12 weeks post‐treatment, however, HFUS grading exceeded clinical grading in 17/30 patients (56.7%), highlighting a divergence in resolution assessment during therapy. HFUS severity scores typically remained higher and resolved more slowly than clinical scores. These findings suggest HFUS provides superior accuracy and objectivity for assessing deep‐seated lesions, offering a more reliable guide for continued medication decisions in later treatment stages. HFUS combined with CDFI offers a promising solution to the aforementioned clinical challenges [15].

Our study demonstrates HFUS's capability to quantify deep‐seated lesions (e.g., nodule/cyst dimensions, depth, dermal thickness, structural characteristics, and fat layer involvement), while CDFI visualizes lesion vascularity. This combined approach enables comprehensive, multidimensional assessment of both deep and superficial pathology. Consequently, HFUS/CDFI represents a valuable tool for managing moderate‐to‐severe acne vulgaris, particularly regarding deep‐seated inflammation and treatment response monitoring [16].

One of the limitations of this study lies in the asymmetry of the assessment units. The HFUS‐SSSA grading system focuses on the structural changes of a single “most severe” target lesion, whereas the Pillsbury grading system evaluates the overall severity of facial acne. This difference in design may lead to reduced agreement between the two methods after treatment (decreased *κ* value). However, this apparent “inconsistency” has important clinical implications, as it highlights the complementary roles of clinical and imaging assessments in capturing different dimensions of acne severity. Pillsbury grading is sensitive to the rapid reduction of superficial lesions, whereas SSSA grading reflects the slower resolution of deep lesions and delayed structural repair.

In clinical practice, this indicates that even when superficial findings meet the criteria for “clinically mild” acne, the persistence of incompletely resolved deep cysts or active blood flow on HFUS (SSSA Grade II–III) suggests residual inflammation and a higher risk of recurrence. Therefore, we recommend a combined use of both methods: the Pillsbury grading system for rapid evaluation and initial treatment guidance, and HFUS/CDFI as a precise monitoring tool for moderate‐to‐severe acne, particularly nodulocystic acne, to assess true resolution of deep inflammation, guide treatment duration, and identify patients at risk of scar formation. This integrative approach may support more individualized and thorough treatment strategies, ultimately reducing recurrence and scar formation rates. Future studies with larger sample sizes may explore the development of composite scoring systems that integrate overall lesion counts with ultrasonographic features of deep lesions to achieve a more comprehensive assessment.

There are additional limitations to this study. Firstly, the sample size was relatively small, and future studies will require larger cohorts. Additionally, limited reference literature exists on HFUS for acne assessment; thus, the accuracy of HFUS evaluations and factors influencing it warrant further investigation.

Recently, Malinowska et al. [17] utilized HFUS to evaluate the efficacy of laser therapy in treating acne scars, affirming HFUS as a valuable tool for both scar assessment and therapeutic monitoring. Furthermore, Nanco‐Melendez et al. [18] applied high and ultra HFUS imaging to compare the treatment outcomes of microneedling versus fractional CO_2_ laser for atrophic facial acne scarring. While these previous studies primarily emphasize the clinical utility of HFUS in evaluating laser‐based interventions for scar improvement, our work distinctly focuses on monitoring the therapeutic response of active inflammatory lesions. Looking forward, we aim to further investigate the potential of ultrasonography in predicting acne scar formation.

In conclusion, the combined HFUS/CDFI approach enables non‐invasive, quantitative assessment of moderate‐to‐severe acne lesions. This facilitates more accurate clinical grading and provides comprehensive monitoring from superficial to deep structures. Consequently, it significantly enhances the evaluation of disease severity, treatment efficacy, and prognosis, thereby reducing recurrence risk. HFUS/CDFI represents a reproducible, efficient tool for acne management.

## Author Contributions

Litao Zheng participated in the writing and revision of the paper. Xiaogang Fu participated in the analysis, interpretation, and data collection. Xiaoying Qiu, Song Gao, Yaqun Jiang, Xue Wang, and Kashif Ali assisted with operation, data collection, and analysis; Fangru Chen supervised the project and finalized the manuscript.

## Funding

This study benefited from funding of the third batch of Lijiang Scholar Award in Guilin (Grant 2022‐5‐07).

## Ethics Statement

This project fully considered and protected the rights and interests of the study objects. It meets the criteria of the Ethical Review Committee. This study has passed ethical review [No. 2021YJSLL‐67]. This study has obtained ethical approval from the Department of Dermatology, the First Affiliated Hospital of Guilin Medical University, 15 Lequn Road, Guilin 541 001, China.

## Consent

All authors approved the final manuscript and the submission to this journal. Written informed consent for publication was obtained from all participants.

## Conflicts of Interest

The authors declare no conflicts of interest.

## Supporting information


**TABLE S1:** Presents the general information on patients with moderate‐to‐severe acne vulgaris.
**TABLE S2:** Summarizes the clinical characteristics of moderate‐to‐severe acne vulgaris.

## Data Availability

The data that support the findings of this study are available from the corresponding author upon reasonable request.
