# Supplementary material for: The Value of High‐Frequency Ultrasound and Color Doppler Flow Imaging in Assessing the Efficacy of Moderate‐to‐Severe Acne Vulgaris: A Prospective Single‐Arm Study
Source: J Cosmet Dermatol. 2026 Mar 31;25(4):e70793. doi: 10.1111/jocd.70793 (PMC13039763; doi:10.1111/jocd.70793)
Supplement: Supplementary file 1 — TABLE S1: Presents the general information on patients with moderate‐to‐severe acne vulgaris. TABLE S2: Summarizes the clinical characteristics of moderate‐to‐severe acne vulgaris. [file JOCD-25-e70793-s001.docx]

**Supplementary Table 1** General information on patients with moderate-to-severe acne vulgaris

|  | | Number of patients (n) | Ratio (%) |
| --- | --- | --- | --- |
| Gender | Male | 14 | 46.7 |
|  | Female | 16 | 53.3 |
| Age at diagnosis, years | <18 | 4 | 13.3 |
|  | 18~25 | 24 | 80.0 |
|  | >25 | 2 | 6.7 |
| Occupation | Students | 23 | 76.7 |
|  | Others | 7 | 23.3 |
| Ethnic group | Han ethnic group | 28 | 93.3 |
|  | Ethnic minority | 2 | 6.7 |
| Educational attainment | Undergraduate and above | 25 | 83.3 |
|  | Junior University | 2 | 6.7 |
|  | High school | 3 | 10.0 |
|  | Junior high school and below | 0 | 0 |
| BMI(kg/m^2^) | <18.5 | 12 | 40.0 |
|  | 18.5~24.9 | 17 | 56.7 |
|  | ≥25 | 1 | 3.3 |

**Supplementary Table 2** Clinical characteristics of moderate-to-severe acne vulgaris

|  | | Number of patients (n) | Ratio (%) |
| --- | --- | --- | --- |
| Site of most severe lesion involvement | Nose and forehead | 5 | 16.7 |
|  | Cheeks | 12 | 40.0 |
|  | Chin | 9 | 30.0 |
|  | Neck | 4 | 13.3 |
| Disease dura-  tion，months | <12 | 4 | 13.3 |
|  | 12~24 | 11 | 36.7 |
|  | >24 | 15 | 50.0 |
| Aggravating season | Spring | 0 | 0 |
|  | Summer | 5 | 16.7 |
|  | Autumn | 1 | 3.3 |
|  | Winter | 2 | 6.7 |
|  | No significant correlation | 22 | 73.3 |
| Skin type | Oil | 23 | 76.7 |
|  | Dry | 0 | 0 |
|  | Mixed | 7 | 23.3 |
|  | Neutral | 0 | 0 |
